# An Urgent Necessity for Clinical Pharmacy Services in Cancer Care in Nepal

**DOI:** 10.1200/GO.20.00434

**Published:** 2020-09-17

**Authors:** Sunil Shrestha, Sujyoti Shakya, Asmita Priyadarshini Khatiwada

**Affiliations:** Sunil Shrestha, PharmD, Department of Pharmacy, Nepal Cancer Hospital and Research Center, Harisiddhi, Lalitpur, Nepal; and Sujyoti Shakya, M Pharm, and Asmita Priyadarshini Khatiwada, M Pharm, Department of Pharmaceutical and Health Service Research, Nepal Health Research and Innovation Foundation, Lalitpur, Nepal

## TO THE EDITOR:

We read the article by Gyawali et al,^1^ entitled “Overview of Delivery of Cancer Care in Nepal: Current Status and Future Priorities,” published in *JCO Global Oncology*. We want to congratulate the authors for this successful article and its contribution to the field of cancer care in Nepal.

Gyawali et al^1^ indicated that the involvement of pharmacists can improve patient cancer care, which we think should be more highlighted. The article reflected the importance of pharmacy services for better patient outcomes and identified pharmacists as the professionals overcoming the gap between oncologists and patients, which we believe is an urgent need in the context of Nepal.^1^ In the case of patients with cancer, clinical pharmacists have an essential function in improving drug use (including chemotherapy and other high-alert medications), reducing adverse effects and toxicity, providing drug education and psychological support, assessing medication response, developing and implementing guidelines on the use and handling of chemotherapeutics, and improving patient outcomes and quality of life. According to Wang et al,^2^ pharmaceutical interventions by clinical pharmacists play a decisive role in enhancing chemotherapy knowledge, improving the patient's positive emotions in dealing with chemotherapy responses, and improving the quality of life. Clinical pharmacists participate in clinical ward rounds in collaboration with medical services specialists and conduct patient interviews, drug reconciliation, and patient guidance and follow-up, which results in enhanced outcomes in oncology settings.^3^

In fact, according to Ma,^4^ the Board of Pharmacy Specialties, an independent division of the American Pharmacists Association, perceives oncology pharmacy as one of the eight specialized fields of pharmacy, with the designation of Board Certified Oncology Pharmacists. The American Society of Clinical Oncology depicts oncology pharmacists as professionals with information on individual anticancer medications and affirms that essential interdisciplinary practice boosts improvement in medication treatment and decreases chemotherapy-related toxicity.

During chemotherapy administration, an oncology pharmacy specialist can examine the patient's hydration resistance, electrolyte abnormalities, potential tumor lysis syndrome, nausea control, vomiting, and other serious adverse effects through patient interviews along with routine monitoring of chemical and vital signs.^4^ Multidisciplinary care teams depend on pharmacists for reliable initial value assessment.^5^ Pharmacists play a unique role in antibiotic selection, dosage, and pharmacokinetic monitoring, especially for patients with fever and neutropenia.^4^ Studies have demonstrated that antibiotic stewardship programs led by pharmacists resulted in a significant decline in usage rates for inappropriate antibiotics, as well as the average duration of treatment. In one study, *Clostridium difficile* infection tripled in the absence of pharmacists from the stewardship team.^6^ Monitoring of patients with genetic variation by a pharmacist can also help prevent potential infectious complications.^7^

A pilot study on the function of clinical pharmacists in a community oncology practice revealed the benefits of an increased role of the clinical pharmacist in identifying adverse drug reactions (particularly chemotherapy-induced nausea and vomiting), decreasing adverse therapeutic outcomes, and improving medication adherence through counseling and regular observation of patients.^8^ Patients found it easier to open up to pharmacists rather than their oncologists about their concerns, and they responded well on telephone follow-up by the pharmacists.^8^ Clinical pharmacists play a crucial part in improving medication management and adherence.

The clinical pharmacist has been involved in compounding chemotherapy agents, among other drugs. The preparation of sterile IV admixtures is an essential and complex part of delivering chemotherapy to patients and is usually performed by nurses or pharmacists, if available. Working pharmacists should carefully manage the appropriate dilution, maintenance of sterility, ventilation, labeling, and dispensing of these drugs.^4^

Clinical pharmacy is a necessary part of oncology care settings and is practiced in many nations. However, clinical pharmacy services are being conducted in a limited number of oncology hospitals in Nepal and are still in the infancy stage. There is much evidence to support the undeniable role the clinical pharmacist can play in patient care. From educating patients regarding their diseases and medications, aiding oncologists and other health care professionals through their expertise on oncology medications, compounding chemotherapy, and developing and implementing guidelines for the preparation and safe handling of chemotherapy, to supportive care for patients facing various consequences of chemotherapy, pharmacists are directly involved in oncology care settings. The integration of clinical pharmacists into oncology settings widely in Nepal can aid in more effective medication management and improve the quality of life for patients with cancer.

## References

[1] Gyawali B Sharma S Shilpakar R et al Overview of delivery of cancer care in Nepal: Current status and future priorities JCO Glob Oncol 6 1211 1217 2020 32735488 10.1200/GO.20.00287 PMC7395484

[2] Wang Y Wu H Xu F Impact of clinical pharmacy services on KAP and QOL in cancer patients: A single-center experience BioMed Res Int 2015 502431 2015 26697487 10.1155/2015/502431 PMC4677164

[3] Kaboli PJ Hoth AB McClimon BJ et al Clinical pharmacists and inpatient medical care: A systematic review Arch Intern Med 166 955 964 2006 16682568 10.1001/archinte.166.9.955

[4] Ma CS Role of pharmacists in optimizing the use of anticancer drugs in the clinical setting Integr Pharm Res Pract 3 11 24 2014

[5] ASPEN Board of Directors et al Guidelines for the use of parenteral and enteral nutrition in adult and pediatric patients JPEN J Parenter Enteral Nutr 26 1sa 138sa 2002 (1 suppl) 11841046

[6] Cappelletty D Jacobs D Evaluating the impact of a pharmacist’s absence from an antimicrobial stewardship team Am J Health Syst Pharm 70 1065 1069 2013 23719885 10.2146/ajhp120482

[7] Moen EL Godley LA Zhang W et al Pharmacogenomics of chemotherapeutic susceptibility and toxicity Genome Med 4 90 2012 23199206 10.1186/gm391 PMC3580423

[8] Dobish R Chambers C Iwaasa K et al Expanding the role of clinical pharmacists in community oncology practice Oncol Exchange 13 24 28 2014

